# Comparison of machine learning models for hemoglobin prediction in patients undergoing maintenance hemodialysis

**DOI:** 10.3389/fmolb.2026.1746108

**Published:** 2026-02-20

**Authors:** Ting Xie, Xiaoyan Su, Chen Yun, Xiaohong Tang, Xuejia Zheng, Jingjing Dong, Qi Guo, Shouping Zhu, Donge Tang, Yong Dai, Lianghong Yin

**Affiliations:** 1 Department of Nephrology, The First Afliated Hospital of Jinan University, Guangzhou, China; 2 Department of Nephrology, Dongguan Tungwah Hospital, Dongguan, China; 3 Charité -Universitätsmedizin Berlin, Berlin, Germany; 4 Guangzhou Institute of Technology, Xidian University, Guangzhou, China; 5 Department of Nephrology, The Second Affiliated Hospital of Guangzhou Medical University, Guangzhou, China; 6 Clinical Medical Research Center, Shenzhen People’s Hospital (The First Affiliated Hospital, Southern University of Science and Technology, The Second Clinical Medical College, Jinan University), Shenzhen, China; 7 Department of General Medicine, Zhejiang Cancer Hospital, Hangzhou Institute of Medicine (HIM), Chinese Academy of Sciences, Hangzhou, China; 8 Department of Materials Science and Engineering, Southern University of Science and Technology, Shenzhen, China; 9 The First Affiliated Hospital, School of Medicine, Anhui University of Science and Technology, Huainan, China; 10 Guangzhou Enttxs Medical Products Co., Ltd., Guangzhou, China

**Keywords:** chronic kidney disease, hemoglobin, machine learning, maintenance hemodialysis, multilayer perceptron

## Abstract

**Objective:**

To estimate the next hemoglobin (Hb) levels in maintenance hemodialysis (MHD) patients, predictive models were developed using various Machine Learning (ML) algorithms.

**Methods:**

A total of 8,159 records from 2,104 MHD patients across 24 blood purification centers in Shenzhen were included. Eight ML algorithms were employed to develop prediction models: Linear Regression (LR), Least Absolute Shrinkage and Selection Operator (Lasso), Bayesian Ridge, Gradient Boosting (XGBoost), Random Forest (RF), Support Vector Machine (SVM), Multilayer Perceptron (MLP), and Long Short-Term Memory (LSTM). Subsequently, the performance of models was evaluated and compared.

**Results:**

Among all the models, the MLP performed the best performance, with an *R* ^2^ of 0.672, a mean absolute error (MAE) of 9.360 g/L, and a root mean square error (RMSE) of 12.438 g/L. The analysis indicated that the most recent Hb value (Hb(t-1)) was the strongest predictor.

**Conclusion:**

ML models based on demographic characteristics, dialysis records, and historical Hb data can effectively predict future Hb levels in MHD patients, which is helpful for early identification of anemia risk and timely clinical intervention.

## 1 Introduction

End stage renal disease (ESRD) is a global public health issue and imposes a large disease burden (Ke et al., 2022). The Global Burden of Disease study estimated the global prevalence of individuals undergoing dialysis to be approximately 3.57 million in 2023 (Collaborators, 2025). As the main renal replacement therapy for ESRD, maintenance hemodialysis (MHD) was used by about 1.027 million patients in China by the end of 2024.

Patients with ESRD often experience renal anemia (RA), with an anemia rate up to 90% (Shen et al., 2021). Numerous studies have shown that RA in MHD patients is associated with various adverse outcomes, such as rapid deterioration of renal function, adverse cardiovascular events, and decline in health-related quality of life (Yang et al., 2020; Toft et al., 2020; Alshogran et al., 2021; Spinowitz et al., 2019). Effective anemia management is thus crucial to improving the prognosis and quality of life in patients receiving MHD.

Machine Learning (ML) is a branch of Artificial intelligence that completes specified tasks through data learning (Borhani et al., 2022). The application of ML in MHD patients is increasing, including the prediction of essential parameters (e.g., blood pressure, dry weight, Kt/V) and the risk of complications such as anemia and mortality (Li et al., 2025; Kim H. R. et al., 2021; Kim H. W. et al., 2021; Kang et al., 2024; Shu et al., 2025). The application of the Anemia Control Model tailored hemoglobin (Hb) target regulation, and was significantly correlated with a lower hospitalization risk in hemodialysis patients (Barbieri et al., 2016; Garbelli et al., 2024). However, existing prediction models are often challenged by inadequate multi-center data standardization and the absence of key clinical variables, which limits their generalizability and clinical applicability.

This study involved the development and comparative evaluation of ML models to predict the next Hb levels in a cohort of MHD patients, utilizing routinely collected data including longitudinal Hb records, dialysis parameters, and demographic characteristics to facilitate personalized anemia management.

## 2 Research methods

### 2.1 Data sources

This study collected 9,619 MHD patients from 44 blood purification centers in Shenzhen from 1 January 2001, to 20 April 2021. During dialysis, patients were dialyzed two or three times a week for 4 hours each time. Blood pressure was collected before, after, and every hour during each dialysis. We excluded patients younger than 18 years old, without Hb test data, hemodialysis record data, and Hb test times <5. Finally, 2104 MHD patients from 24 blood purification centers were included as the final research objects of this study. This study was approved by the Ethics Committee of Shenzhen People’s Hospital (LL-KY-2021, 870), Shenzhen, China.

### 2.2 Data preprocessing and feature selection

Hb(t-4), Hb(t-3), Hb(t-2), Hb(t-1), and Hb(t) from five consecutive tests at any time during dialysis were selected. Hb(t) was defined as the prediction target, namely, the Hb value of the next detection, and Hb at other times was used as the model features. Then the blood pressure from t-1 to t in dialysis records was processed to obtain new model features, where variation was defined as the difference between the actual value and the mean value. The patient’s age, gender, systolic blood pressure, diastolic blood pressure and their related features, as well as five consecutive Hb values, were recorded as one piece of data. To clarify, intervals between consecutive Hb measurements were variable (due to individualized follow-up schedules) and not incorporated into our data structure. Table 1 provided a detailed description of the definitions and assignment instructions for the included features. SPSS 23.0 statistical software was used for basic data analysis. The measurement data of normal distribution was described as mean ± standard deviation, and the counting data was described as number of cases and percentage. Detailed basic information of data record features were shown in Table 2.

**TABLE 1:** The definition of features.

Features	Definition	Value
Age	Age	Quantitative value
Gender	Gender	Male = 1; female = 0
SBP_mean	Mean of systolic blood pressure	Quantitative value
SBP_std	Standard deviation of SBP	Quantitative value
SBP_diff_abs_mean	Absolute mean of SBP variation	Quantitative value
SBP_diff_abs_std	Absolute standard deviation of SBP variation	Quantitative value
SBP_diff_mean	Mean of SBP variation	Quantitative value
SBP_diff_std	Standard deviation of SBP variation	Quantitative value
DBP_mean	Mean of diastolic blood pressure	Quantitative value
DBP_std	Standard deviation of DBP	Quantitative value
DBP_diff_abs_mean	Absolute mean of DBP variation	Quantitative value
DBP_diff_abs_std	Absolute standard deviation of DBP variation	Quantitative value
DBP_diff_mean	Mean of SBP variation	Quantitative value
DBP_diff_std	Standard deviation of SBP variation	Quantitative value
MEAN_AP_mean	Mean of mean arterial pressure	Quantitative value
MEAN_AP_std	Standard deviation of mean arterial pressure	Quantitative value
IDH	Intradialytic hypotension	exist = 1; no = 0
Hb(t-4)	Prior Hb of Hb (t-3)	Quantitative value
Hb(t-3)	Prior Hb of Hb (t-2)	Quantitative value
Hb(t-2)	Prior Hb of Hb (t-1)	Quantitative value
Hb(t-1)	Prior Hb of Hb(t)	Quantitative value
Hb(t)	Target Hb	Quantitative value

**TABLE 2:** Detailed basic information of data record features.

Features	Data record (n = 8159)
Age (year)	54.02 ± 16.16
Male (n, %)	4871, 59.70%
SBP_mean (mmHg)	142.12 ± 24.26
SBP_std (mmHg)	10.40 ± 6.56
SBP_diff_abs_mean (mmHg)	11.09 ± 7.43
SBP_diff_abs_std (mmHg)	6.84 ± 5.44
SBP_diff_mean (mmHg)	1.09 ± 6.39
SBP_diff_std (mmHg)	11.75 ± 8.64
DBP_mean (mmHg)	80.85 ± 15.61
DBP_std (mmHg)	6.02 ± 7.90
DBP_diff_abs_mean (mmHg)	6.71 ± 10.73
DBP_diff_abs_std (mmHg)	4.24 ± 7.60
DBP_diff_mean (mmHg)	0.05 ± 7.23
DBP_diff_std (mmHg)	7.23 ± 11.45
MEAN_AP_mean (mmHg)	101.27 ± 16.86
MEAN_AP_std (mmHg)	6.80 ± 6.23
IDH (1, %)	2256, 27.65%
Hb(t-4) (g/L)	96.25 ± 22.26
Hb(t-3) (g/L)	96.76 ± 22.18
Hb(t-2) (g/L)	97.38 ± 21.81
Hb(t-1) (g/L)	98.07 ± 21.50
Hb(t) (g/L)	98.69 ± 21.80

Feature selection was performed based on the comparative results of root mean square error (RMSE) against the number of features for Random Forest (RF) and Elastic Net models. The predictive accuracy of candidate models was evaluated using RMSE, a key metric for Hb levels. After excluding Hb(t) as the prediction target, 21 candidate features were retained and utilized to develop all predictive models. Following the principle of minimizing prediction error, the combination of features with the lowest error rates were selected and subsequently incorporated into all ML models for Hb prediction.

### 2.3 Model selection and performance evaluation

We assessed and compared the performance of multiple modeling algorithms, including linear models (Linear Regression (LR), Least Absolute Shrinkage and Selection Operator (Lasso), and Bayesian Ridge), ensemble methods (such as Gradient Boosting (XGBoost) and RF), high-dimensional or nonlinear models (e.g., Support Vector Machine (SVM)), and deep learning architectures (including Multilayer Perceptron (MLP) and Long Short-Term Memory (LSTM)). Data analysis was conducted using the ML algorithm library Scikit-learn 1.0.2 implemented in Python (Pedregosa et al., 2011). Model transparency was presented in Supplementary Material.

To ensure the reliability of model performance estimation, the dataset was partitioned via a stratified 80:20 hold-out split into a training subset and an independent test subset. Hyperparameter tuning was conducted using five-fold cross-validation restricted to the training subset, thereby avoiding data leakage between the training and test partitions. The generalization ability of the final tuned model was assessed on the independent test set using the coefficient of determination (*R* ^2^), mean absolute error (MAE), and RMSE. RMSE was set as the primary evaluation metric. The benchmark model was defined as the one with the lowest RMSE. To compare the predictive performance of all models with that of the benchmark model, the normality of RMSE differences was first assessed via the Shapiro-Wilk test. Paired t-tests with Bonferroni correction were applied for pairwise comparisons if the RMSE differences conformed to a normal distribution; otherwise, the Wilcoxon signed-rank test was employed.

## 3 Results

### 3.1 Basic information

**FIGURE 1:**
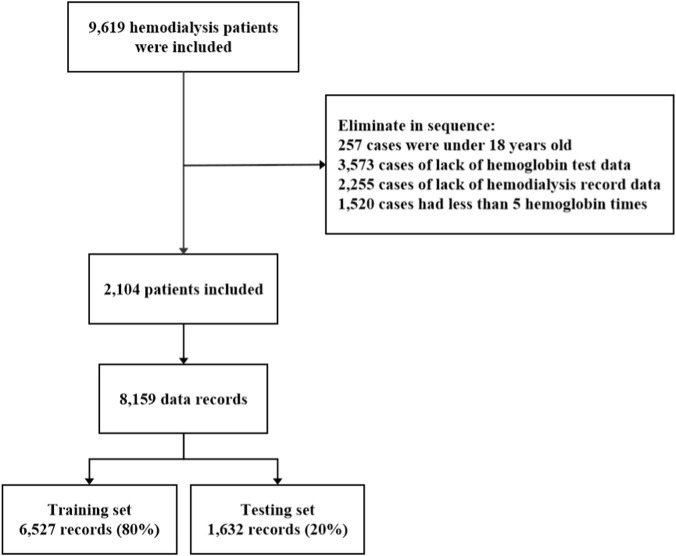
Flowchart of patient enrollment, screening, and dataset partitioning. Among 9,619 patients initially assessed, 2,104 were eligible for inclusion, generating 8,159 records that were allocated to training (80%) and test (20%) datasets.

**FIGURE 2:**
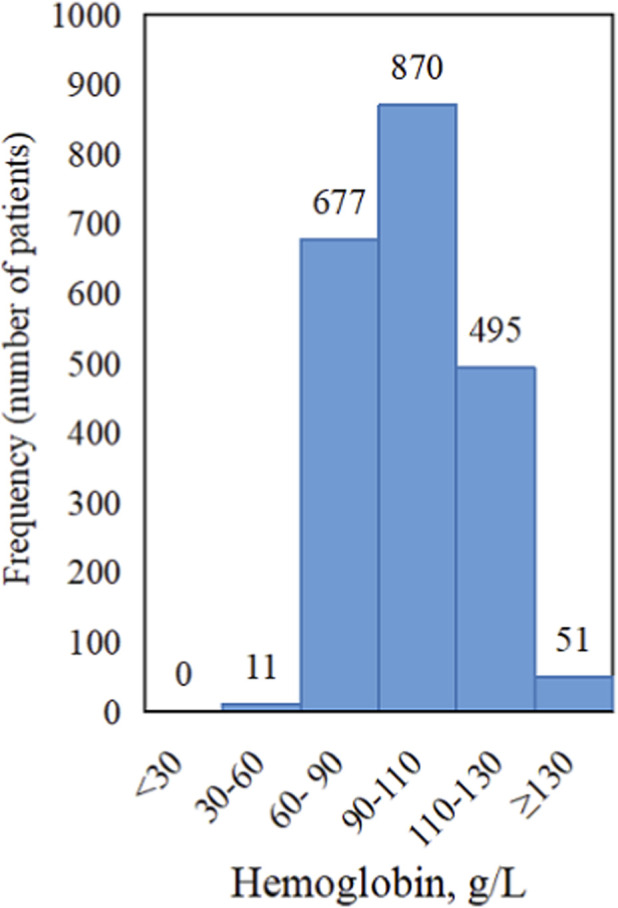
Frequency distribution of hemoglobin (Hb) levels in the study population (n = 2,104). The majority of observations were within the range of 60–130 g/L.

We enrolled 2,104 MHD patients from 24 blood purification centers in Shenzhen, resulting in a final dataset of 8,159 records (Figure 1). The average age of patients participating in the study was 54.02 ± 16.16 years old, and male accounted for 59.70%. The mean Hb level during the observation period was 97.97 ± 16.70 g/L, with the majority of patients exhibiting values within the range of 60–130 g/L (Figure 2). The detailed information of the features in the data record was shown in Table 2.

### 3.2 Feature selection and SHAP analysis

**FIGURE 3:**
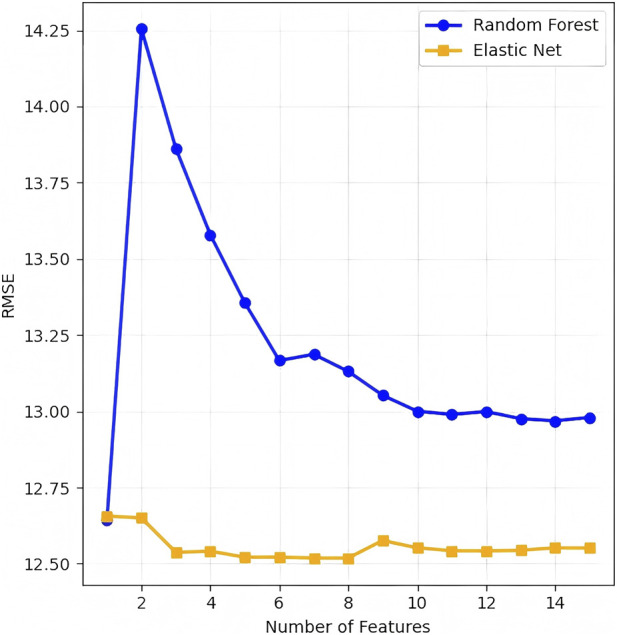
Feature selection optimization. Root mean square error (RMSE) values for the Random Forest and Elastic Net models demonstrated that optimal model performance plateaus at 6 features.

**FIGURE 4:**
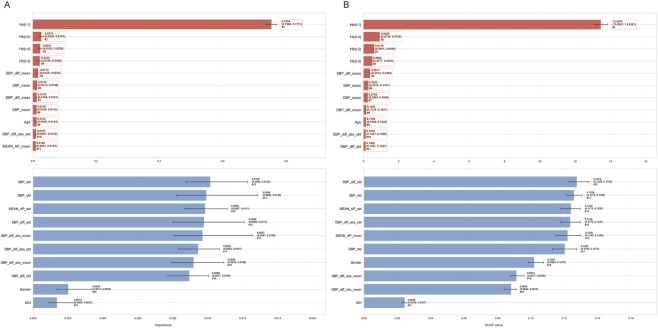
Model interpretability analysis **(A)** Feature importance ranking based on Random Forest and **(B)** SHAP values identifying Hb(t-1) and SBP parameters as the strongest predictors.

Based on the patterns of RMSE in relation to the number of features for the RF and Elastic Net models, the RMSE values of both models reached or approached their minimum levels when the feature number was set to 6 (Figure 3). The top 6 predictors identified by the RF model were as follows: Hb(t-1), Hb(t-2), Hb(t-4), Hb(t-3), SBP_diff mean, and SBP mean (Figure 4A). These selected features were defined as the final predictive feature set and incorporated into all subsequent ML models for Hb levels prediction. The top 6 features identified by SHAP analysis were fully consistent with the top 6 features ranked by RF feature importance, reinforcing that these covariates were the most critical drivers of Hb levels prediction (Figure 4B).

### 3.3 Model performance comparison

**FIGURE 5:**
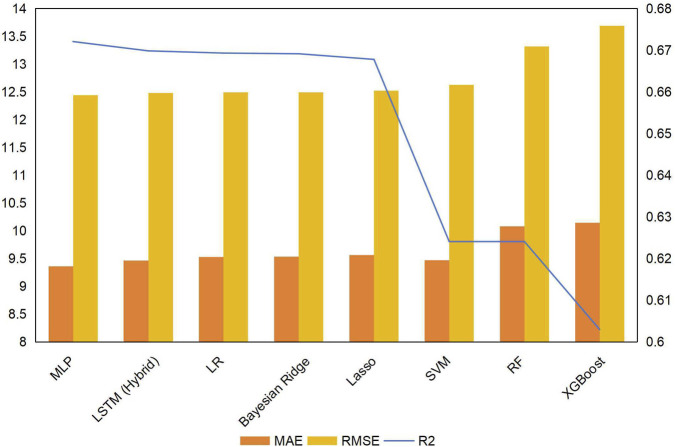
Performance comparison of eight machine learning models. The Multilayer Perceptron (MLP) model achieved significantly superior predictive performance, with a coefficient of determination (*R* ^2^) of 0.672 and a root mean square error (RMSE) of 12.438.

Using the top 6 features as input, we built 8 ML models with their respective algorithms. *R* ^2^, MAE, and RMSE, as performance evaluation indexes of prediction models were shown in Table 3 and Figure 5. The MLP model achieved the highest *R* ^2^ value and the lowest MAE and RMSE among all candidate models. Statistical analysis using paired t-tests with Bonferroni correction further confirmed that the MLP model exhibited significantly superior predictive accuracy compared to all other models (P < 0.05), based on differences in RMSE. These findings collectively indicated that the MLP model demonstrated superior accuracy and clinical applicability for predicting Hb levels in MHD patients.

**TABLE 3:** Performance evaluation indexes of prediction models using the top 6 features.

Models	*R* ^2^	MAE	RMSE	Comparison with MLP
MLP	0.672053	9.360747	12.438173	-
LSTM (Hybrid)	0.669824	9.464916	12.480358	0.006446
LR	0.669291	9.526813	12.490429	0.001586
Bayesian Ridge	0.669124	9.532605	12.493586	0.001727
Lasso	0.667777	9.563647	12.518990	0.003220
SVM	0.624077	9.470317	12.625002	0.003840
RF	0.624077	10.080685	13.316931	0.000295
XGBoost	0.602982	10.142349	13.685471	0.000556

MLP, multilayer perceptron; LR, linear regression; Lasso, Least absolute shrinkage and selection operator; SVM, support vector machine; RF, random forest; XGBoost, Gradient Boosting; *R* ^2^, the coefficient of determination; MAE, mean absolute error; RMSE, root mean squared error.

To verify whether the models outperform simple linear fitting and avoid overestimating performance caused by the autoregressive effect of Hb(t−1), we constructed an LR model using the same features. Compared with LR, MLP achieved an *R* ^2^ of 0.672, demonstrating a slight but stable improvement. This result indicated that beyond the strong autoregressive effect of Hb(t−1), MLP could capture the subtle non-linear associations between features and Hb levels that cannot be resolved by linear fitting.

In addition, the predictive performance of all models was evaluated using the full feature set for comparison (Table 4). Our results demonstrated that predictive performance was consistently improved across all models when using the top 6 features, relative to the complete feature set.

**TABLE 4:** Performance evaluation indexes of prediction models using the full features.

Models	*R* ^2^	MAE	RMSE
LR	0.669374	9.511276	12.488872
Bayesian Ridge	0.667977	9.539164	12.515222
Lasso	0.665798	9.563185	12.556220
LSTM (Hybrid)	0.655486	9.552188	12.748474
RF	0.647083	9.687298	12.903015
SVM	0.646450	9.821718	12.914571
XGBoost	0.595570	10.390483	13.812622
MLP	0.106594	10.145912	20.529531

LR, linear regression; Lasso, Least absolute shrinkage and selection operator; LSTM, Long Short-Term Memory; RF, random forest; SVM, support vector machine; XGBoost, Gradient Boosting; MLP, multilayer perceptron; *R* ^2^, the coefficient of determination; MAE, mean absolute error; RMSE, root mean squared error.

## 4 Discussion

Traditional statistics primarily aims to characterize relationships between data and outcome features through statistical inference, typically utilizing complete datasets for this purpose. In contrast, ML focuses on achieving precise predictions by optimizing model performance on independent test datasets, without requiring prior assumptions about functional relationships between predictors (X) and outcome (Y). This methodology systematically divides the dataset into training and test subsets, specifically designated for model development and validation purposes, respectively.

This study utilized a comprehensive dataset collected from 24 blood purification centers to develop prediction models for the next Hb levels in MHD patients. The MLP model achieved superior predictive accuracy in our analysis, which aligned with previous research employing ML for Hb prediction in similar patient populations (Martinez-Martinez et al., 2014; Barbieri et al., 2015).

Consistent with previous finding (Martinez-Martinez et al., 2014), the most recent Hb value Hb(t-1) was identified as the strongest predictor of Hb(t), with importance significantly outperforming other clinical and demographic features. In patients undergoing regular MHD without acute blood loss, Hb levels generally remain stable over the lifespan of red blood cells and more directly reflect the current hematologic status. In contrast, earlier historical values (e.g., Hb(t-2) to Hb(t-4)) demonstrate diminished predictive power as they may exceed this physiological timeframe. Furthermore, studies have shown that the Hb level of patients with RA gradually increases over time after treatment (Coyne et al., 2022). Since Hb(t-1) is the measurement value that is closest to the target detection time and within the lifespan of red blood cells, it can more effectively reflect the cumulative effect of continuous treatment. Therefore, compared with other previous Hb values, Hb(t-1) has greater predictive value.

The present study demonstrated that blood pressure-related parameters (SBP_diff mean, and SBP mean) hold significant predictive value for Hb levels. Although the current observational predictive model data has not yet been confirmed, these associations may have reasonable physiological pathways as a basis. However, there are still only hypotheses that need to be evaluated. Higher blood pressure variability may induce compensatory erythropoiesis via vascular remodeling and subsequent tissue hypoxia (Intengan and Schiffrin, 2001; D’Alessandro and Xia, 2020), while hypertension can indirectly exacerbate anemia by triggering renal inflammation and fibrosis—pathological processes that impair erythropoietin synthesis and upregulate hepcidin expression (Cui et al., 2017). Collectively, these observations indicate that blood pressure parameters are correlated with Hb levels through the aforementioned mechanistic pathways, and the predictive value of such parameters in our machine learning models is thus underpinned by a well-established clinicopathological rationale.

Notably, age and gender were not included in the final predictive feature set, as they ranked low in feature importance and their independent contribution to Hb levels prediction was significantly weaker than that of historical hemoglobin values and blood pressure-related parameters; their incorporation would not effectively improve the predictive performance of the model. This finding was consistent with the clinical characteristics of the MHD population, in which the inherent physiological effects of age and gender on Hb levels are masked by strong clinical interventions, including regular dialysis and standardized management of renal anemia, thus reducing their relative predictive value in this specific cohort.

The innovative aspect of this study lies in its development and systematic benchmarking of eight distinct ML algorithms for predicting subsequent Hb levels in MHD patients, utilizing a large-scale, multi-center clinical dataset from 24 blood purification centers. Contrasting with previous studies limited to single-center data or restricted features selection, our approach integrated longitudinal dialysis records with multi-timepoint laboratory data, which significantly improved the generalization ability and clinical application potential of the model, and provided a reliable data-driven tool for individualized anemia management.

This study has several limitations. First, as an observational investigation, it cannot establish causal relationships between the selected features and Hb levels; future cohort or experimental studies are needed to elucidate the underlying mechanisms. Second, this study did not conduct subgroup analysis. Subsequent work can reduce population heterogeneity through grouping to enhance the reliability of the conclusion. Another limitation of this study is that the variable time interval for Hb measurements may compromise the interpretability and reproducibility of the models. Furthermore, the lack of external/temporal validation in this study may have weakened the broad applicability of the model. In addition, due to the inconsistent progress and standards of electronic information system construction among various blood purification centers, some early data were recorded in paper form, resulting in limited scale and insufficient completeness of electronic data, such as the lack of detailed medication records, blood transfusion history, iron metabolism indicators, and dialysis doses and other key features. After the information system is improved, more features can be incorporated to optimize the model’s prediction performance.

## 5 Conclusion

This study demonstrated that demographic characteristics, dialysis treatment records, and historical Hb data serve as effective predictors of Hb levels in maintenance MHD patients. Among all evaluated ML models, the MLP exhibited optimal performance. Our research provides a novel perspective on anemia management strategies from a non-pharmacological intervention standpoint, although its clinical applicability requires further validation. Notably, the early prediction of Hb levels using ML models enables clinicians to assess patients’ anemia status ahead of routine blood tests. This not only provides a theoretical basis for improving the individualized and precise management of anemia in dialysis patients but also creates practical possibilities for improving the long-term prognosis of patients.

## Data Availability

The raw data supporting the conclusions of this article will be made available by the authors, without undue reservation.
