# Supplementary material for: Comparison of machine learning models for hemoglobin prediction in patients undergoing maintenance hemodialysis
Source: Front Mol Biosci. 2026 Feb 20;13:1746108. doi: 10.3389/fmolb.2026.1746108 (PMC12962952; doi:10.3389/fmolb.2026.1746108)
Supplement: Supplementary file 1 [file Supplementaryfile1.docx]

**Supplementary Material**

1. Detailed Clarification of the Hybrid Architecture and Its Distinctions from the Standard LSTM.

The LSTM (hybrid type) model in this study is not the conventional single-input LSTM; instead, it is a dual-input hybrid neural network that integrates:

A temporal branch to process sequential historical hemoglobin values (Hb(t-1), Hb(t-2), ...).

A static feature branch to process non-temporal clinical indicators (e.g., age, sex, other laboratory parameters).

A fusion layer that combines the outputs of both branches before the final prediction.

This design enables the model to simultaneously capture temporal dynamics and static patient-specific factors, which is clinically relevant for hemoglobin prediction.

Comparison between Standard LSTM and LSTM (Hybrid)

| Aspect | Standard LSTM | LSTM(Hybrid) |
| --- | --- | --- |
| Input Type | Single sequential input | Dual inputs: sequential + static |
| Input Shape | [samples, timesteps, features] | Two separate inputs:  1. [samples, timesteps, 1] (temporal)  2. [samples, n_static_features] (static) |
| Feature Fusion | None (single modality) | Concatenation after LSTM layer |
| Parameter Count | Relatively lower | Higher (due to additional dense layers for static features) |
| Clinical Rationale | Purely temporal forecasting | Multi-modal: captures both trends and static covariates |

1. Model transparency: Hyperparameters, MLP architecture, and LSTM configuration.

Hyperparameter details are shown in the code. The specific information of the MLP architecture and LSTM configuration is as follows:

MLP_ARCHITECTURE = {

'hidden_layer_sizes': (100, 50),

'activation': 'relu',

'solver': 'adam',

'alpha': 0.0001,

'batch_size': 'auto',

'learning_rate': 'constant',

'learning_rate_init': 0.001,

'power_t': 0.5,

'max_iter': 500,

'shuffle': True,

'random_state': RANDOM_STATE,

'tol': 0.0001,

'early_stopping': True,

'validation_fraction': 0.1,

'beta_1': 0.9,

'beta_2': 0.999,

'epsilon': 1e-08,

'n_iter_no_change': 10,

#others

'momentum': 0.9,

'nesterovs_momentum': True,

'warm_start': False,

'max_fun': 15000 }

LSTM_CONFIG = {

'sequential_features_pattern': 'Hb(t-*',

'scaler_static': StandardScaler(),

'scaler_sequential': StandardScaler(),

'input_sequential_shape': {

'timesteps': None,

'features': 1

},

'input_static_shape': {

'features': None

},

'lstm_layer': {

'units': 50,

'activation': 'relu',

'recurrent_activation': 'sigmoid',

'use_bias': True,

'kernel_initializer': 'glorot_uniform',

'recurrent_initializer': 'orthogonal',

'bias_initializer': 'zeros',

'unit_forget_bias': True,

'dropout': 0.0,

'recurrent_dropout': 0.0,

'return_sequences': False,

'return_state': False,

'go_backwards': False,

'stateful': False,

'unroll': False

},

'dense_layers': [

{

'units': 50,

'activation': 'relu',

'kernel_initializer': 'he_normal',

'use_bias': True

},

{

'units': 1,

'activation': None,

'kernel_initializer': 'glorot_uniform',

'use_bias': True

}

],

'concatenation_layer': {

'axis': -1

},

'training': {

'optimizer': {

'type': 'adam',

'learning_rate': 0.001,

'beta_1': 0.9,

'beta_2': 0.999,

'epsilon': 1e-07,

'amsgrad': False

},

'loss': 'mse',

'metrics': ['mae', 'mse'],

'epochs': 100,

'batch_size': 32,

'validation_split': 0.2,

'shuffle': True,

'early_stopping': {

'monitor': 'val_loss',

'min_delta': 0.001,

'patience': 10,

'verbose': 1,

'mode': 'min',

'restore_best_weights': True

},

'callbacks': [

EarlyStopping(

monitor='val_loss',

patience=10,

restore_best_weights=True

)

],

'verbose': 0,

'class_weight': None,

'sample_weight': None,

'initial_epoch': 0

},

'compile': {

'optimizer': 'adam',

'loss': 'mse',

'metrics': ['mae']

}

}
